# Temperature-Sensitive Fragrance Microcapsules with Double Capsule Walls: A Study on Preparation and Sustained Release Mechanism

**DOI:** 10.3390/polym15183686

**Published:** 2023-09-07

**Authors:** Qun Yang, Genghao Hu, Huili Qiu, Rajib Mia, Hongjuan Zhang, Liujun Pei, Jiping Wang

**Affiliations:** 1 School of Textiles and Fashion, Shanghai University of Engineering Science, Shanghai 201620, China; 2 Shanghai Engineering Research Center for Clean Production of Textile Chemistry, Shanghai 201620, China

**Keywords:** fragrance microcapsules with double capsule walls, poly-n-isopropyl acrylamide, temperature responsiveness, sustained release

## Abstract

Microcapsules are small particles that can effectively protect a core material from degradation. Microcapsules with double capsule walls can improve stability and reduce breakage due to the fact that the physical and chemical properties of double-walled materials can complement each other, thus enhancing the quality and applicability of a microcapsule. Microcapsules can achieve controlled release of core materials by using a temperature-sensitive wall material. In this research, gelatin was used as the inner wall material for these double-walled microcapsules. The outer wall material was a composite material prepared by the reaction of a hydroxyl group in gum arabic with an amino group in N-isopropylacrylamide (NIPAM) in the presence of N, N’-methylene bisacrylamide (BIS), while lavender fragrance oil served as the core material. A complex coalescence method was used for the preparation of microcapsules with double capsule walls. The effects of different proportions of gum arabic to NIPAM on the core loading, microcapsule yield and thermal stability of microcapsules were studied in detail. Additionally, the stability of these fragrance microcapsules with double capsule walls in different solvents and pH values was evaluated. The sustained release properties and mechanism of cotton fabrics treated with prepared fragrance microcapsules were investigated. The results show that the microcapsules prepared with a 10:1 ratio of NIPAM to gum arabic have good temperature responsiveness. Therefore, clothing treated with microcapsules with temperature-sensitive wall materials can ensure that the human body has a fresh and pleasant smell in the case of perspiring in summer.

## 1. Introduction

Microencapsulation is an advanced technology that involves the encapsulation of tiny droplets or particles of a liquid or solid substance within a continuous film made of a polymeric material. This technique is used to encapsulate substances that are sensitive to environmental factors such as light, heat and moisture. There are various substances that can be encapsulated using this technique, for example, fragrance oil, pharmaceuticals, enzymes and food ingredients [1,2,3]. Capsulation can reduce the loss of core materials, facilitate storage, reduce oxidation, improve shelf life, etc. However, there are challenges associated with the application of microcapsules, including lack of flexibility, easy breaking and the limited range of suitable encapsulant materials, which result in a short residence time of the fragrance oil, poor binding with textiles and lack of resistance to water washing. These problems make it difficult to achieve the desired effect. To solve these problems, various approaches have been adopted, including selecting suitable wall materials, modifying and assembling wall materials, modifying the manufacturing processes of microcapsules and designing new structures [4,5]. Microcapsules with double walls have been receiving special attention because the physical and chemical properties of the two layers can complement each other and thus synergistically improve their quality [6]. Some common wall materials used for microencapsulation include natural, biodegradable polymers such as gelatin, gum arabic, chitosan, maltodextrin, starch, carboxymethyl cellulose, etc. During the microencapsulation process, the choice of wall material has an important influence on the stability and release performance of the microencapsulation process.

In recent years, there has been a growing interest in environmentally responsive microcapsules [7]. These microcapsules are designed to control the release of their core material by changing their physical and chemical properties in response to changes in the external environment such as temperature, pH, light, moisture, magnetic field and electric fields, etc. Environmentally responsive microcapsules are widely used in drug delivery, biosensing, electrocatalysis and enzyme immobilization [8,9]. Temperature-dependent microcapsules typically contain temperature-sensitive polymers in their capsule walls that undergo reversible phase transitions at the lower critical transition temperature (LCST). This results in changes in the properties of the capsule wall [10,11,12]. Poly(N-isopropylacrylamide (PNIPAM) is a temperature-sensitive polymer that can be synthesized from N-isopropylacrylamide (NIPAM) via free-radical polymerization. It is easy to functionalize. The LCST of PNIPAM is approximately 32 °C, which is close to the normal human body temperature. Therefore, microcapsules made with PNIPAM as the wall material can be used for applications such as drug delivery and bionic materials. However, due to poor biodegradability, its application in humans is limited. Nevertheless, its application in vitro, such as the application in slow-release smart clothing, shows promise [13,14,15].

Complex coalescence is a microencapsulation technique that involves the interaction of oppositely charged polyelectrolytes in an aqueous medium. Due to the electrostatic attraction between the two biopolymers, this process leads to the formation of condensation or precipitation [16]. Complex coacervation is a highly promising microencapsulation technique that is extensively employed in various industries such as pharmaceutical, food, agriculture and textile industries. This technique has several advantages, including high encapsulation efficiency, low cost and ease of operation [17,18].

Fragrance not only has a calming effect on the mind and body (lavender fragrance oil), but also can repel mosquitoes and other insects (peppermint essential oil) [19]. At the same time, fragrance also reflects the cultivation and taste of the wearer. Textiles with embedded fragrance microcapsules are called aromatherapeutic textiles. Such textiles provide the scent of fragrance oils derived from microcapsules which can boost emotional and physical characteristics of the body. Fragrance released from the textile by breaking the microcapsule shell can improve the volatilization of the fragrance oil and achieve sustained fragrance release. Microcapsules can be applied to textiles by impregnation, spraying or coating. Microcapsules can release fragrance over a long period of time in various textile applications [20].

Therefore, in this work, a complex coalescence method was used to prepare temperature-sensitive fragrance microcapsules with double capsule walls. Gelatin was used as the inner wall material, while a composite material prepared with gum arabic and N-isopropylacrylamide in the presence of BIS was used as the outer wall material. The stability of the prepared fragrance microcapsules under different solvents and pH values was investigated. The sustained release properties and fragrance oil release behaver of cotton fabrics treated with the prepared fragrance microcapsules were studied. Through this research, it is expected to solve the problems of low flexibility and easy breakage of traditional microcapsules, while imparting temperature sensitivity to achieve the controlled release of fragrance oil, which can ensure that the human body has a fresh and pleasant smell in the case of perspiring in summer or for use in curtains, bedding accessories and carpets.

## 2. Materials and Methods

### 2.1. Experimental Materials

Gum arabic, lavender fragrance oil, sodium dodecyl sulfate and gelatin were purchased from SinoPharm Chemical Reagent Co., Ltd. (Shanghai, China). N-isopropylacrylamide (NIPAM), ammonium persulphate (APS), N, N′-methylene bisacrylamide (BIS) and ethanol were purchased from Titan Technology Co., Ltd. (Shanghai, China). These are analytical reagents and were used as received without further purification. Maleic-methyl acrylate copolymer and cotton fabric (5 cm × 5 cm, 140 g/m^2^) were provided by Shanghai Yichun Industrial Co., Ltd. (Shanghai, China).

### 2.2. Temperature-Sensitive Composite Wall Material Preparation

**Scheme 1:**
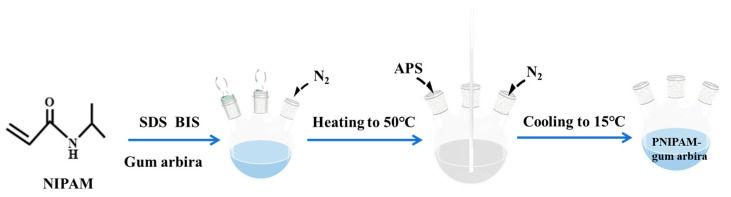
Preparation process of temperature-sensitive composite wall materials.

Temperature-sensitive composite wall materials were prepared using gum arabic and N-isopropyl acrylamide (NIPAM) as raw materials in the presence of BIS. The 1% concentration of gum arabic was stirred at a constant temperature of 50 °C until it became homogeneous. NIPAM, aqueous gum arabic, sodium dodecyl sulfate (SDS) and BIS were then dissolved in deionized water. Nitrogen was placed in deionized water at 50 °C for 1 h to remove air. Then, 0.05 g ammonium persulfate (APS) was added to initiate polymerization and the product was cooled to room temperature after 4 h. The cooled product was placed in molecular weight cut-off (MWCO, 800–1400 Da) dialysis bags for a week to remove unreacted monomers and oligomers. The water was changed 2–3 times a day to ensure that the unreacted monomers and oligomers were completely removed from the product. Five different ratios (0:1, 5:1, 10:1, 15:1 and 20:1) of NIPAM to gum arabic were prepared, as shown in Table 1. The preparation process is shown in Scheme 1.

### 2.3. Preparation of Fragrance Microcapsules with Double Capsule Walls

Gelatin aqueous solution with 1% concentration was stirred at 50 °C. Then, the emulsifier (8% to lavender fragrance oil), lavender fragrance oil and gelatin solution (2% to lavender fragrance oil) were mixed. The mixed solution was homogenized and emulsified at a speed of 5000 rpm for 35 min to form a uniform emulsion. The emulsion was then stirred at a certain speed while the prepared temperature-sensitive composite wall materials were slowly added. The two polyelectrolytes were combined by interionic forces and coalesced to form microcapsules with double capsule walls.

### 2.4. Aromatic Fabric Prepared with Fragrance Microcapsules

The dipping method is a simple and easy-to-operate technique. In this research, an upright resin press machine was used to make the finishing liquid pass through the fiber gap by roll pressure. The samples were dipped and padded twice, and the pick-up rate of liquid was 120%.

### 2.5. Characterizations

#### 2.5.1. Structural Analysis

FTIR spectra were collected by a Fourier transform spectrophotometer (NEXUS 670, Thermo Nicolet Corporation, Madison, Wisconsin, USA) at room temperature in the region of 500–4000cm^−1^ with a spectral resolution of 2 cm^−1^ and 32 scans, which can be used to characterize the chemical composition of materials. Optical microscopy, scanning electron microscopy (SEM) and transmission electron microscopy (TEM) were used to observe the microstructure of microcapsules. A small amount of microcapsule emulsion was dipped onto a microscope slide and evenly coated on the cover glass. Its microstructure was observed using a polarizing microscope (Shanghai Optical Instrument Factory, Shanghai, China). Temperature-sensitive fragrance microcapsules with double capsule walls were dispersed in anhydrous ethanol and the microscopic morphology was observed by a Hitachi S4800 scanning electron microscope (Hitachi, Tokyo, Japan). The acceleration voltage was 20 kV. The surface morphology of cotton fabric and aromatic cotton fabric prepared by fragrance microcapsules was also analyzed by SEM. The temperature-sensitive fragrance microcapsules with double capsule walls were dripped onto a copper mesh to naturally dry into a film, and the bilayer structure of the microcapsules was observed with TEM from Japan Electron Corporation (Tokyo, Japan).

#### 2.5.2. Thermogravimetric Analysis and Differential Scanning Calorimetry Analysis

The thermal stability of temperature-sensitive wall materials, temperature-sensitive fragrance microcapsules with double capsule walls, fragrance microcapsules with single capsule walls and lavender fragrance oil were analyzed using a TGA55 thermogravimetric analyzer (PerKin Elmer Pyris, Waltham, MA, USA) in the range of 25–700 °C under N_2_ protection at a flow rate of 20 mL/min. The heating rate was 20 °C/min. Differential scanning calorimetry (DSC) analysis of temperature-sensitive wall materials was performed using a differential scanning calorimeter (PerKin Elmer Pyris, Waltham, MA, USA) in the range of 5–50 °C with a heating rate of 5 °C/min under the N_2_ atmosphere at a flow rate of 20 mL/min.

#### 2.5.3. Yield and Encapsulation Efficiency of Microencapsulation

Microcapsule yield (MY) is the ratio of the mass of solid microcapsules obtained to the total mass of core and wall materials added [21]. The yield of microcapsules can reflect the utilization rate of the wall and core materials during the preparation. This is calculated with the following equation:

(1) $$\mathrm{MY}=\frac{W_{1}}{W_{2}}\times 100\%$$

where *W*_1_ is the mass of solid microcapsules after freeze-drying (g); *W_2_* is the total mass of wall and core materials added (g).

Encapsulation efficiency (LC) is the percentage of the mass of essential fragrance oil encapsulated in the microcapsules to the whole microcapsules, which can visually reflect the encapsulation capacity of the microcapsules [22]. A small amount (about 1 g) of prepared temperature-sensitive fragrance microcapsules with double capsule walls was added into a Petri dish. Then, it was dried at 50 °C in a vacuum oven for 24 h. LC was calculated using the following equation:

(2) $$\mathrm{LC}\%=1-\frac{\left(M_{1}-M_{2}\right)-\beta \left(M_{1}-M_{0}\right)}{\alpha \left(M_{1}-M_{0}\right)}\times 100\%$$

where α represents the mass fraction of the core material to the sample microencapsulated emulsion, β represents the mass fraction of volatile components other than fragrance oil to the sample microencapsulated emulsion, M_0_ is the mass of the empty dish (g), M_1_ is the mass of the fragrance microcapsule and dish (g), and M_2_ is the mass of solid microcapsules after drying (g). 

#### 2.5.4. Stability Testing

The stabilities of fragrance microcapsules at different pH values and different solvents were investigated. The pH of the microcapsule emulsion was adjusted to different values of 3–12 for acid–base stability testing. Ethylene glycol, methanol, acetone, isopropanol and ethanol solutions were added into the microcapsule emulsion, respectively, for solvent stability testing. Both acid–base stability and solvent stability of the fragrance microcapsules were investigated by visual observation, optical microscopy and gas chromatography-mass spectrometry (GC-MS). 

#### 2.5.5. Sustained Release Properties and Mechanism Analysis

The contents of fragrance oil in cotton fabrics after treatment with fragrance microcapsules were quantified and analyzed. Headspace gas chromatography was used to analyze the fragrance oil release properties. The peak areas of the signature substances were used to calculate the content of fragrance oil. In the chromatogram, the peak with high intensity and significant variation over time was selected. Gas chromatographic conditions were as follows: column of 30 m in length, 0.25 mm in diameter, 0.25 μm in film thickness and 30 kPa in column pressure. The carrier gas used in the testing was 99.995% pure helium and the flow rate was set at 1 mL/min. The temperature was from 60 °C to 200 °C at a rate of 10° C/min and held for 5 min, then increased up to 280 °C at a rate of 30 °C/min and held for 5 min. The injector temperature was maintained at 250 °C, the electron energy was 700 eV and the mass scan range was 35–650 amu. Two parallel samples were prepared and analyzed. The peak areas of fragrance oil for different natural days were measured. The release was expressed as the percentage of the residual content of fragrance oil using the following equation:

W = *C_i_*/*C*_0_ = *A_i_*/*A*_0_ (3)

where *C_i_* represents the concentration of the measured component in the specimen after being placed at different times. *C*_0_ represents the concentration of the initially estimated component in the model. *A_i_* represents the peak area of the measured part in GC-MS after being placed for different periods of times. *A*_0_ represents the peak area of the initial component in GC.

## 3. Results and Discussion

### 3.1. Structure and Characteristics of Temperature-Sensitive Composite Materials

**Figure 1:**
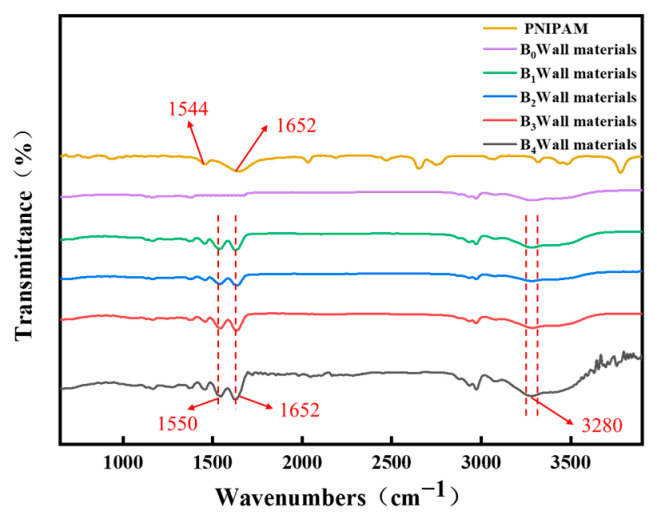
FTIR spectra of temperature-sensitive wall materials.

Figure 1 shows the FTIR spectra of different wall materials. Absorption peaks of the amide group of PNIPAM were found at 1544 cm^−1^ and 1652 cm^−1^. Wall materials of B_1_, B_2_, B_3_ and B_4_ were observed at absorption peaks of 1652 cm^−1^, but the characteristic peak at 1544 cm^−1^ was shifted to 1550 cm^−1^ due to the addition of gum arabic. The absorption peak of the amide group of B_0_ was not detected, as the addition amount of NIPAM was 0. The absorption peaks at 3280 cm^−1^ of B_1_, B_2_, B_3_ and B_4_ were the characteristic peaks of hydrogen bonds, which were formed by the free hydrogen in -CO-NH- from NIPAM with the hydrogen in the -OH in gum arabic [23,24]. Since the polymerization of NIPAM with BIS takes place in the presence of gum arabic, there is the possibility for radical trapping by the hydroxyl groups of the gum arabic, thus grafting the PNIPAM to the gum arabic. This indicates that the temperature-sensitive composite had been successfully prepared using gum arabic and NIPAM as raw materials.

**Figure 2:**
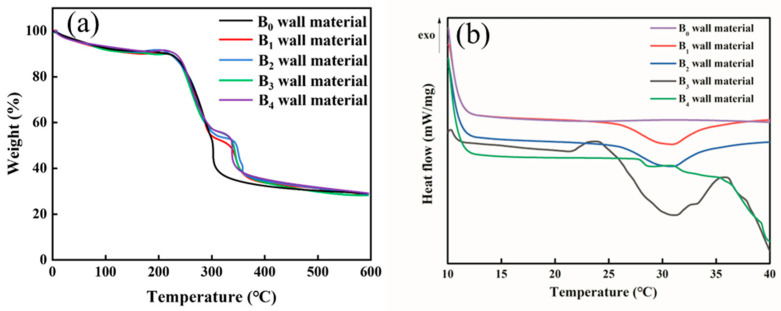
TG (**a**) and DSC (**b**) curves of temperature-sensitive wall materials.

TG and DSC curves of the temperature-sensitive composite materials are shown in Figure 2. From Figure 2a, the thermal weight loss of B_0_ with 0 NIPAM was that of gum arabic. Its thermal degradation mainly consists of two stages. The weight loss below 100 °C was mainly due to the loss of water in gum arabic. The second weight loss at 200–450 °C was the thermal degradation of gum arabic. Comparing B_1_, B_2_, B_3_ and B_4_ with B_0_, the thermal degradation consists of three stages. After the added NIPAM, the second weight loss was between 200 and 320 °C and the third weight loss was from 320 to 400 °C. In the third weight stage, the weight loss was about 20%, mainly from PNIPAM by thermal decomposition, which is also consistent with previous studies [25].

As shown in Figure 2b, the DSC curve of the gum arabic (B_0_) and the temperature-sensitive wall materials (B_1_, B_2_, B_3_ and B_4_) with the addition of NIPAM were significantly different. The range of phase transition of B_1_, B_2_, B_3_ and B_4_ was significantly more extensive with the addition of NIPAM. This also indicated the difference in the internal structure and chemical composition of the temperature-sensitive wall material [26]. In addition, the variation in heat flux of B_2_ (10:1) was more prominent than that of other wall materials. Also, the latent heat of B_2_ was more obvious and had better thermal stability. All these results indicated that the composite wall materials had been successfully prepared.

### 3.2. Structure, Yield and Encapsulation Efficiency of Temperature-Sensitive Fragrance Microcapsules with Double Capsule Walls

The yield and encapsulation efficiency of the temperature-sensitive fragrance microcapsules with double capsule walls with different composites as the outer wall materials are shown in Table 2. Different temperature-sensitive wall materials influenced the microcapsules’ outcome and encapsulation efficiency. The yield of B_0_ as the outer wall material was the highest: it was up to 78 ± 0.57%. In contrast, the yield and encapsulation efficiency of B_1_, B_3_ and B_4_ as the outer wall materials were decreased due to the change in the outer capsule wall of the microcapsules from gum arabic to the composite wall materials made by the polymerization of NIPAM and gum arabic. Unsuitable composite walls made from different proportions of NIPAM and gum arabic may suffer from polymerization failure, breakage, deformation, etc., which can cause the escape of fragrance oil encapsulated by single-wall materials. Meanwhile, the interaction of oppositely charged polyelectrolytes of gum arabic to gelatin in aqueous solutions may decrease after the addition of the NIPAM molecular structure in gum arabic. The encapsulation efficiency and yield of B_2_ as the outer wall material were almost the same as that of B_0_. This further indicated that B_2_ was a suitable microcapsule wall material, which is consistent with the conclusions from DSC.

**Figure 3:**
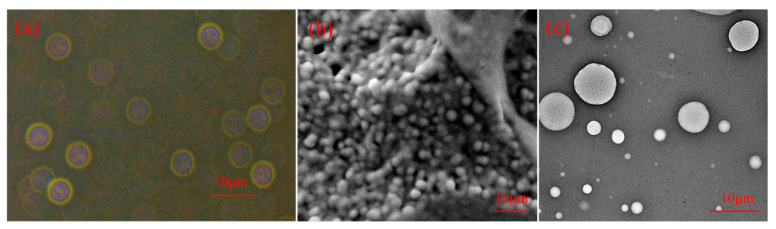
(**a**) Optical microscope image; (**b**) SEM image and (**c**) TEM image of temperature-sensitive microcapsules with double walls.

The microstructures of temperature-sensitive fragrance microcapsules with double capsule walls were observed using an optical microscope, SEM and TEM. The images are shown in Figure 3a–c, respectively. In Figure 3a,b, it can be seen that the microcapsules were spherical in shape with a smooth surface and have uniform particle size. The internal fragrance oil and bilayer structure can be seen clearly in Figure 3a. In Figure 3c, it can be clearly seen that the bilayer structure of the microcapsules and the fragrance oil is completely encapsulated. The bilayer structure as well as the homogeneity of the double-encapsulated microcapsules are illustrated, which accords with the experimental expectation.

**Figure 4:**
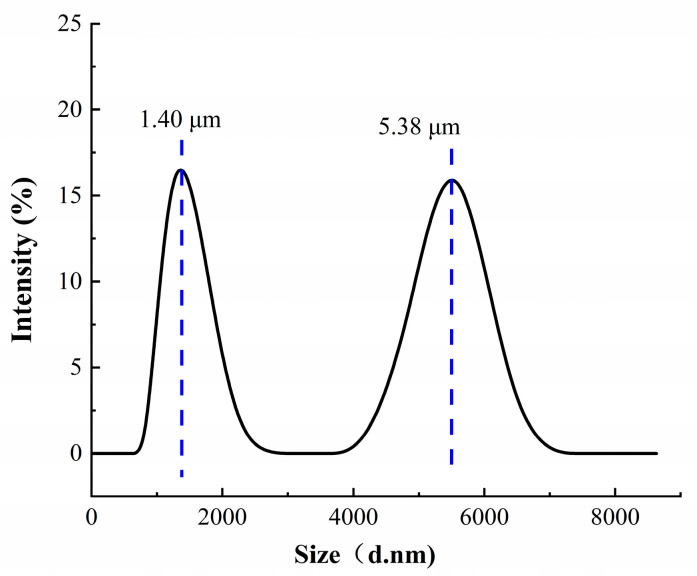
Diameters and distributions of temperature-sensitive microcapsules with double walls.

The diameters and distributions of the temperature-sensitive fragrance microcapsules with double capsule walls are shown in Figure 4. From the figure, it can be seen that the first fraction appears as a shoulder to the fraction centered at ca. 1.40 μm but is well-separated, and the second fraction appears as a shoulder to the fraction centered at ca. 5.38 μm. From the TEM images in Figure 3c, it can be seen that there are particles with different sizes. The bimodal particle size distribution is usually revealed by TEM images [27]. The mean diameter can be calculated by fitting the Gaussian function.

**Figure 5:**
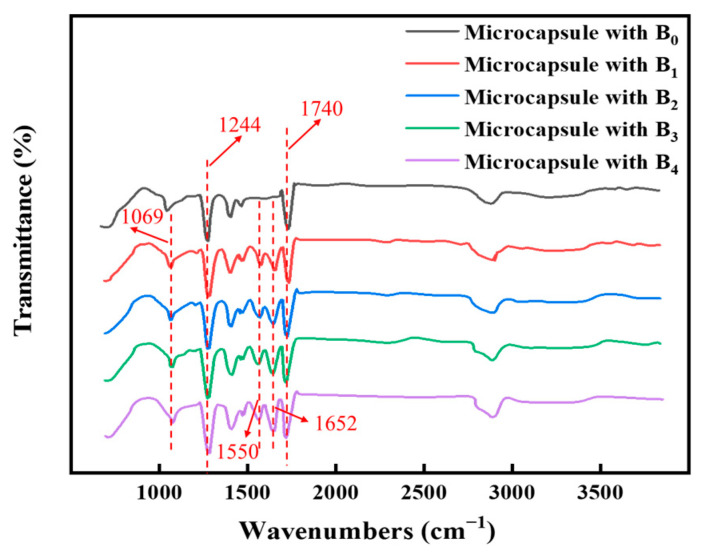
FTIR spectra of temperature-sensitive microcapsules with double walls.

Figure 5 shows the FTIR spectra of the temperature-sensitive fragrance microcapsules with different capsule walls. The characteristic peaks of B_1_, B_2_, B_3_ and B_4_ as microcapsule wall materials were found at 1652 cm^−1^ and 1550 cm^−1^. These were the distinct peaks of amide groups. The characteristic peaks of lavender fragrance oil were found in the infrared spectra of all five ratios of temperature-sensitive microcapsules, such as 1740 cm^−1^ and 1244 cm^−1^. The absorption peak of gelatin was shifted to 1069 cm^−1^, which was due to the complex coalescence reaction between gelatin and the temperature-sensitive wall material.

### 3.3. Thermal Performance of Temperature-Sensitive Fragrance Microcapsules with Double Capsule Walls

**Figure 6:**
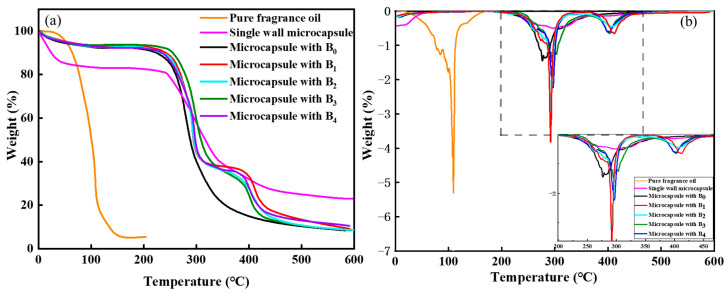
TG (**a**) and DTG (**b**) of temperature-sensitive microcapsules with double walls compared with single-wall microcapsule and pure fragrance oil.

Figure 6 shows the TG curves of the temperature-sensitive fragrance microcapsules with double capsule walls using different temperature-sensitive composite materials (B_1_, B_2_, B_3_ and B_4_, respectively) and the material with 0 NIPAM addition (B_0_). The TG curves of the fragrance microcapsule with a single-encapsulated wall and pure fragrance oil are also shown in Figure 6. The loss rate of lavender fragrance oil in the fragrance microcapsules with double capsule walls was significantly reduced due to the protection of the double-encapsulated wall. For B_0_ microcapsules, weight loss was divided into two main stages. Weight loss that occurred below 100 °C was mainly from the sample’s loss of moisture. Weight loss between 100 and 270 °C was primarily due to the release of lavender fragrance oil from the microcapsules, with a weight loss of about 35%. In this temperature range, the covalent bonds formed by electrostatic forces between gelatin and gum arabic were broken. The lavender fragrance oil encapsulated in the gelatin and gum arabic was continuously escaping. The weight loss between 270 and 450 °C was due to the thermal degradation of the microcapsule wall materials, including gum arabic and gelatin. The weight loss reached about 50% at this stage. As shown in Figure 6, the fragrance oil escape temperatures of B_1_, B_2_, B_3_ and B_4_ were slightly increased compared to B_0_. For B_1_, B_2_, B_3_ and B_4_, weight loss was divided into three main stages. The temperature of the weight loss in the second stage was much higher than that of fragrance microcapsule with a single encapsulated wall (Figure 6b). This indicates that the composite wall material has better heat resistance than the single gum arabic wall material, so that fragrance oil can be better protected. In addition, for B_1_, B_2_, B_3_ and B_4_, the third weight loss of about 20% occurred at 320–450 °C. This was due to the thermal decomposition of the polymer PNIPAM gum arabic in the wall material [28]. This was also consistent with previous discussions.

### 3.4. Stability of Temperature-Sensitive Fragrance Microcapsules with Double Capsule Walls

**Figure 7:**
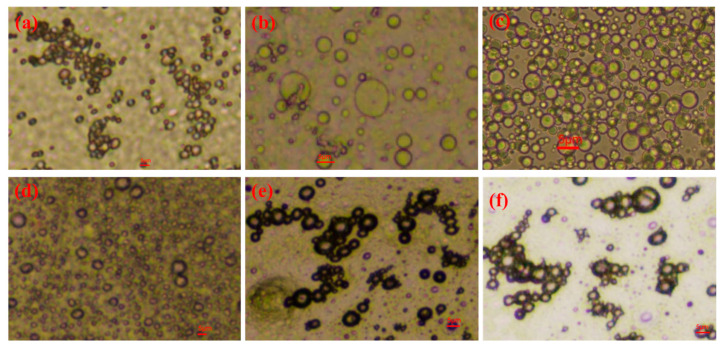
Morphology of microcapsules at different pH: (**a**) pH = 3; (**b**) pH = 5; (**c**) pH = 7; (**d**) pH = 9; (**e**) pH = 11 and (**f**) pH = 12.

Figure 7 shows the microscopic observations of emulsions of fragrance microcapsules with double capsule walls at different pH values. When the external ambient temperature was lower than 32 °C and the pH value of the emulsion was 3–5, there was obvious coalescence in the microcapsule emulsion, because at this pH value the microcapsule emulsion was strongly acidic. Acids can act as hydrogen bond donors and acceptors. Therefore, the hydrogen bonds already formed by PNIPAM and gum arabic were broken. The force between PNIPAM and water molecules was weakened, so the stability of the emulsion was reduced and the microcapsules in the emulsion ruptured to a severe extent.

When the pH value was 7–9, hydrogen bonds were formed between PNIPAM and water molecules. This is because the amide groups in PNIPAM can act as hydrogen bond acceptors, while the water molecules can act as hydrogen bond donors. The hydrophilic effect caused by the hydrogen bonding causes PNIPAM to be fully swollen in water. The molecular chains were stretched and the polymer dispersion stability was excellent. When the pH value was 11–12, the emulsion was strongly alkaline. The hydrogen bonds between PNIPAM and water molecules were broken. This is because the high pH causes the amide groups in PNIPAM to deprotonate and become negatively charged. The negative charges repel the water molecules and reduce the hydrogen bonding [15]. The hydrophobicity between PNIPAM and water molecules was higher than the hydrophilicity. This means that PNIPAM prefers to avoid contact with water and forms a collapsed state. Therefore, the emulsion stability was reduced and dehydration precipitation occurred.

**Figure 8:**
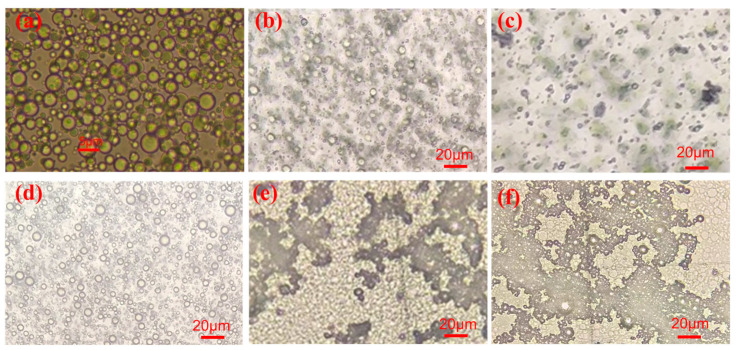
Microcapsule emulsions after solvent addition at room temperature: (**a**) blank (no solvent addition); (**b**) methanol; (**c**) ethanol; (**d**) acetone; (**e**) isopropyl alcohol and (**f**) ethylene glycol.

Figure 8 shows the microscopic observation of emulsions of fragrance microcapsules with double capsule walls under different solvents. The microcapsule emulsions showed different degrees of agglomeration after adding the solvents. In particular, after the addition of isopropyl alcohol, ethylene glycol and ethanol, the morphology of the microcapsules changed significantly and the agglomeration was apparent.

**Figure 9:**
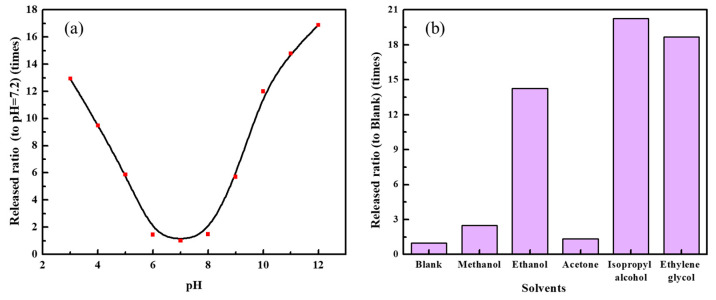
Fragrance oil content under different pH (**a**) and under different solvents (**b**).

To further confirm the effect of pH change and different solvents on microcapsule stability, GC-MS was used to measure the released fragrance oil content of the microcapsule emulsions of the temperature-sensitive fragrance microcapsules with double capsule walls. The fragrance oil content released from the temperature-sensitive fragrance microcapsules with double capsule walls at a pH value of 7 was specified as the blank control, and the ratio of the released fragrance oil of other microcapsule emulsions at different pH values to this control is shown in Figure 9a. The content of fragrance oil released from the temperature-sensitive double-encapsulated wall microcapsules without an added solvent was specified as a blank control, and the ratio of the released fragrance oil of other microcapsule emulsions at different pH solvent values to this control is shown in Figure 9b. In Figure 9a, it can be seen that when the pH value of the emulsions gradually decreases from 7 to 3, the amount of fragrance oil released from the temperature-sensitive fragrance microcapsules increases from 1.45 times (pH = 6) to 12.94 times (pH = 3). Because the microcapsule emulsions coagulated, the microcapsules ruptured to different degrees at acid conditions and the fragrance oil encapsulated was continuously released. Similarly, as the pH of the emulsions gradually increased from 7 to 12, the microcapsules coalesced and ruptured, and the fragrance oil was released from 1.48 (pH = 8) times to 16.92 times (pH = 12). In summary, when the pH of the microcapsule emulsions was in the range of 6–9, there was only a slight difference in the fragrance oil contained. This indicated that the microcapsules were less ruptured. The microencapsulated emulsions were more stable at these pH values. 

In Figure 9b, we can see that the ratios of the released fragrance oil after adding methanol and acetone to the blank (no solvent) were changed less: 2.48 times and 1.35 times, respectively. This indicated that methanol and acetone had less of an effect on the stability of the microcapsule emulsions. After adding isopropyl alcohol, ethylene glycol and ethanol, the ratios of the released fragrance oil changed to 20.28 times, 18.70 times and 14.25 times, respectively. This was due to the reaction of the -CO-NH- of PNIPAM in the outer wall material with the -OH from isopropyl alcohol, ethylene glycol and ethanol. The outer capsule wall ruptured and the fragrance oil encapsulated in it was released. The released fragrance oil content was 14–20 times that of the blank sample, which indicated that isopropyl alcohol, ethylene glycol and ethanol had a significant influence on the stability of the temperature-sensitive fragrance microcapsules with double capsule walls.

### 3.5. Microstructure, Sustained Release Property and Mechanism Research

**Figure 10:**
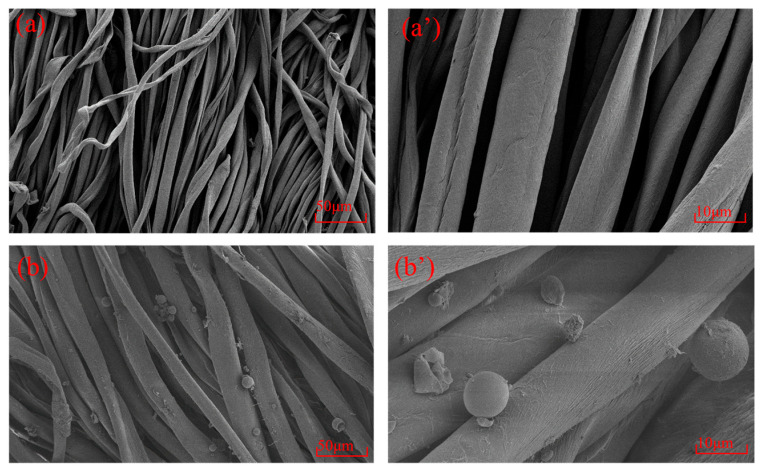
SEM images of cotton fabric (**a**,**a’**) and cotton fabric treated with temperature-sensitive fragrance microcapsules with double capsule walls (**b**,**b’**).

SEM was used to investigate the microstructure of cotton fabrics before and after treatment with microcapsules, and the results are shown in Figure 10. The SEM images show the spherical morphology and smooth surface of the microcapsules on cotton fibers. 

**Figure 11:**
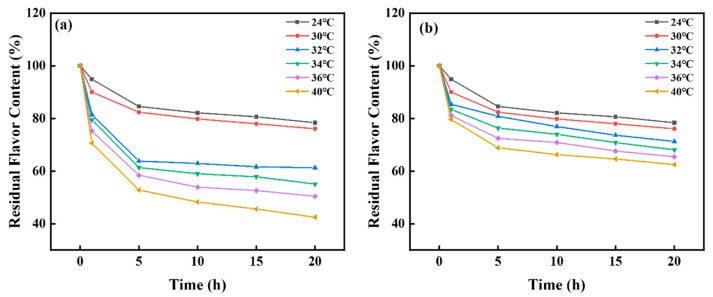
Fragrance oil content of cotton fabrics at different temperatures: (**a**) cotton fabrics treated with temperature-sensitive microcapsule with double walls; (**b**) cotton fabrics treated with microcapsule with double walls (without temperature sensitivity).

Figure 11 shows the results of GC-MS, which was used to determine the fragrance oil content in cotton fabrics. Cotton fabrics were treated with the fragrance microcapsules with double capsule walls (B_0_ as the outer wall material without temperature sensitivity) and the temperature-sensitive fragrance microcapsules with double capsule walls (B_2_ as the outer wall material) with the pad–dry–cure method, respectively. Aromatic cotton fabrics were placed in an oven at 24 °C, 30 °C, 32 °C, 34 °C, 36 °C and 40 °C for 0 h, 1 h, 5 h, 10 h, 15 h and 20 h, respectively. As seen in Figure 11a, which illustrates the sustained release performance of the cotton fabrics treated with the temperature-sensitive fragrance microcapsules with double capsule walls, the fragrance oil was volatilized slowly at 24 °C and 30 °C. After 20 h, the fragrance oil content was about 75.0%, while when the fabrics were at 32 °C, 34 °C, 36 °C and 40 °C, the amount of fragrance oil in cotton fabrics decreased. The higher the temperature, the faster the rate of volatilization of fragrance oil. After 20 h, the fragrance oil contents in the cotton fabrics were 61.3%, 55.1%, 50.4% and 42.5%, respectively. This was because when the external environment temperature reached the LCST of PNIPAM (which is around 32 °C), the hydrogen bonds between the polymer molecules were weakened and the conformation of the molecular structure changed and began to shrink and curl, turning into a random hematic shape, which led to the collapse of the polymer chains and the formation of aggregates [25]. Thus, the outer capsule wall became porous and released the encapsulated fragrance oil. However, since the microcapsules were double walled, there was still an inner wall to protect the encapsulated fragrance oil. Therefore, the sustained release performance of the microcapsules was still better than that of the pure fragrance oil. Figure 11b illustrates the sustained release performance of cotton fabrics treated with ordinary fragrance microcapsules with double capsule walls without temperature sensitivity. After 20 h, the fragrant oil content was retained at over 60%, and there was no sudden release of fragrance oil after the temperature exceeded 32 °C. Therefore, the temperature-sensitive wall material caused the temperature-sensitive release.

The data in Figure 11a were used to substitute into models for correlation fitting to investigate the release performance of the cotton fabric treated with the temperature-sensitive fragrance microcapsules with double capsule walls. The models include the following.

The zero-order release model (zero-order) [29]:

Mt/M_∞_ = k × t + k_0_ (4)

The first-order release model (first-order):

ln(1 − Mt/M_∞_) = k × t + k_0_ (5)

The Higuchi release model [30]:

M_t_/M_∞_ = k × t_1/2_ + k_0_ (6)

**Figure 12:**
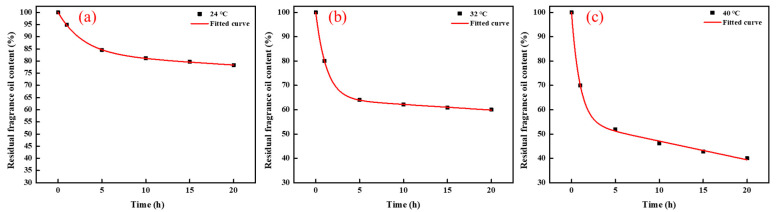
Fragrance oil content in cotton fabrics at different temperatures: (**a**) first-order model at 24 °C; (**b**) first-order model at 32 °C; (**c**) first-order model at 40 °C.

It was found that the release curves of the fabrics treated with the temperature-sensitive fragrance microcapsules with double capsule walls at 24 °C, 32 °C and 40 °C are consistent with the first-order release model. The results are shown in Figure 12. Figure 12a shows the fitted curve of the first-order release model at 24 °C. The release equation is

y = 17.3182 × exp(−x/2.8636) + 0.42 × (−x/1.9266) + 86.7465(7)

where the correlation coefficient R^2^ = 0.9994. 

At 24 °C, the release rate of the fragrance oil was slower: the fragrance oil release amount was about 15.5% in 5 h. The release rate gradually decreased with the time extension, and the cumulative release was 20.7% at 20 h.

When the fabric was placed at 32 °C (LCST of PNIPAM), the first-level release equation of the material was

y = 35.5246 × exp(−x/1.232) + 0.2638 × (−x/1.1477) + 64.4701(8)

where the correlation coefficient R^2^ = 0.9997, as shown in Figure 12b.

At the beginning, the fragrance oil was suddenly released from the fabric: the release amount was 36.1% within 5 h. Due to the molecular structure of PNIPAM and gum arabic, since the polymerization of NIPAM with BIS takes place in the presence of gum arabic, there is the possibility for radical trapping by the hydroxyl groups of the gum arabic, and thus grafting the PNIPAM to the gum arabic. When the external environment reached the LCST of PNIPAM (around 32 °C), the hydrogen bonds formed between NIPAM and gum arabic were broken, because PNIPAM contained both hydrophobic isopropyl groups and hydrophilic amide groups in its structure. The hydrophobic isopropyl groups are responsible for the hydrophobic interactions, while the hydrophilic amide groups are responsible for the hydrogen bonding between the polymer chains and water molecules. This caused the molecules to curl into an irregular cluster structure, and the microcapsules exhibited a double-walled structure. The inner capsule wall still protected the encapsulated fragrance oil, and the release of the temperature-sensitive fragrance microcapsules with double capsule walls was similar to that of the microcapsules with single capsule walls. Hence, its release performance was still better than the pure fragrance oil. After that, the fragrance oil released slowly: only about 1% of release occurred in 15–20 h.

When the fabric was placed at 40 °C (higher than the LCST of the temperature-sensitive double-encapsulated wall microcapsules), the release equation was

y = 45.1997 × exp(−x/0.9658) + 0.869 × (−x/1.1312) + 54.7822(9)

where the correlation coefficient R^2^ = 0.9992, as shown in Figure 12c. 

At the temperature higher than the LCST, the release amount was up to 30.1% in 1 h at 40 °C, which was comparable to the release amount of fabric in 5 h at 32 °C. The remaining fragrance oil in fabrics decreased exponentially in 5 h: it was only 49.2%. This was not only due to the phase change of the outer temperature-sensitive capsule wall, but also because the increase in temperature would accelerate the fragrance oil release, which made fragrance oil releasing at 40 °C more than that at 32 °C. Finally, the accumulated release in 20 h was about 61.3%. In summary, the fabric treated with the temperature-sensitive fragrance microcapsules with double capsule walls showed a good temperature responsiveness. Cotton fabric treated with the temperature-sensitive fragrance microcapsules had controlled release with temperature sensitivity.

## 4. Conclusions

In this paper, temperature-sensitive fragrance microcapsules with double capsule walls were prepared by a complex coalescence method using gelatin as the inner wall material and composite materials obtained from NIPAM and gum arabic as the outer wall materials. The effects of different proportions of gum arabic to NIPAM on core loading, microcapsule yield and thermal stability of the prepared temperature-sensitive fragrance microcapsules with double capsule walls were investigated. A microscopic morphological analysis revealed that the microcapsules were spherically shaped, had smooth surfaces and had uniform particle size and bilayer structure. Considering the encapsulation efficiency, yield and thermal stability of microcapsules, the optimal ratio of NIPAM to gum arabic was 10:1. The results of the stability of temperature-sensitive fragrance microcapsules with double capsule walls showed that the microcapsules’ emulsion was stable at a pH value of 6–9. The addition of the acetone solution had less effect on the stability. In addition, the cotton fabrics treated with the temperature-sensitive fragrance microcapsules with double capsule walls showed significant differences in fragrance oil release below and above 32 °C. In comparison, the fragrance oil was released at a higher rate when the temperature was above the LCST of the wall material, which showed good temperature responsiveness. Therefore, this technology can be used to ensure that the human body has a fresh and pleasant smell in the case of perspiring in summer or can be used in curtains, bed sheets and carpets.

## Figures and Tables

**Table 1:** Five different composite wall materials.

Sample	NIPAM (g)	Gum Arabic (g)	SDS (g)
B_0_	0	0.1	0.05
B_1_	0.5	0.1	0.05
B_2_	1.0	0.1	0.05
B_3_	1.5	0.1	0.05
B_4_	2.0	0.1	0.05

**Table 2:** Yield and encapsulation efficiency of five different composites as the outer wall materials.

Sample	Yield (%)	Encapsulation Efficiency (%)
B_0_	78 ± 0.57	59 ± 1.06
B_1_	68 ± 0.63	52 ± 1.14
B_2_	74 ± 0.79	61 ± 1.12
B_3_	71 ± 0.45	55 ± 1.12
B_4_	65 ± 0.89	53 ± 1.31

## Data Availability

Not applicable.
